# Diabetes and NAFLD: A Synergistic Threat to Metabolic Health

**DOI:** 10.34172/apb.025.44094

**Published:** 2025-10-18

**Authors:** Shilpa Chaudhary, Keerti Manocha, Praveen Malik, Monica Aggarwal, Rekha Rao, Minakshi Garg

**Affiliations:** ^1^School of Pharmaceutical Sciences, Delhi Pharmaceutical Sciences and Research University, Pushp Vihar Sector 3, New Delhi- 110017, India; ^2^ESIC Medical College & Hospital, NH-3, NIT, Faridabad-121001, India; ^3^Department of Pharmaceutical Sciences, Guru Jambheshwar University of Science & Technology, Hisar 125001, India

**Keywords:** Non-alcoholic fatty liver disease, Type 2 diabetes mellitus, Antidiabetic medications

## Abstract

Nonalcoholic fatty liver disease (NAFLD) and type 2 diabetes (T2Dm) are increasingly recognized as interrelated metabolic disorders, each contributing to the other’s progression. NAFLD, a leading cause of chronic liver disease globally, is often underdiagnosed due to its asymptomatic nature. The startlingly high frequency of NAFLD, especially in those with T2Dm, emphasizes the need of thorough screening in high-risk groups. In the setting of T2Dm, the pathophysiology of NAFLD comprises intricate metabolic pathways that exacerbate the disease’s progression. These pathways include insulin resistance, lipotoxicity, and chronic inflammation. Early diagnosis and timely intervention are crucial to prevent the advancement of NAFLD to more severe stages, such as nonalcoholic steatohepatitis (NASH) and cirrhosis. Current guidelines advocate for routine NAFLD screening in patients with T2Dm, emphasizing the importance of early detection. Therapeutic approaches have evolved that are pivotal in managing these intertwined conditions. Each of these treatments offers unique benefits, from improving glycemic control to mitigating liver fat accumulation and reducing cardiovascular risks. This review highlights the pathophysiological linkages, clinical implications, and therapeutic advancements in managing these conditions. By exploring global prevalence, emerging diagnostic tools, and novel therapies, we propose an integrative framework for improved patient outcomes.

## Introduction

 Nonalcoholic fatty liver disease (NAFLD) includes a spectrum of liver conditions in which excessive fat accumulates in the liver (steatosis) of individuals who consume little or no alcohol. This spectrum includes nonalcoholic steatohepatitis (NASH), marked by inflammation and liver cell damage, and simple fatty liver (NAFL), which shows minimal or no inflammation or damage. NASH increases the risk of liver failure, cirrhosis, and fibrosis.^1,2^ The disease can progress from steatosis to cirrhosis, often with periods of stability or regression.

 Clinicians diagnose NAFLD by detecting steatosis in more than 5% of hepatocytes on histology or by measuring liver fat content above 5.6% using proton magnetic resonance spectroscopy (¹H-MRS).^3^ NAFLD has become a leading cause of hepatocellular carcinoma (HCC) and now ranks among the primary indications for liver transplantation in Western countries.^4,5^ Rising obesity rates, particularly in rural areas, along with sedentary lifestyles and poor diets linked to urbanization, have driven this trend.^6,7^ The prevalence of NAFLD also increases with age, especially among postmenopausal women.^8^

 Treatment primarily aims to reduce cardiovascular (CV) and liver-related mortality. Clinicians monitor intermediate outcomes, such as hepatic fibrosis and steatosis, to track disease progression. Although many individuals with hepatic steatosis remain complication-free, some progress to NASH, which can advance to cirrhosis and HCC.^9^ Early diagnosis and management of diabetes-associated NAFLD play a crucial role in preventing progression and related complications.^10^ In 2023, experts revised the NAFLD nomenclature to “metabolic dysfunction-associated steatotic liver disease (MASLD)” to improve awareness and reduce stigma associated with terms like “fatty liver” and “nonalcoholic.”^11^ However, this review uses term NAFLD, as the American Diabetes Association (ADA) has not yet formally adopted MASLD.

 NAFLD often coexists with type 2 diabetes mellitus (T2DM), metabolic syndrome (MetS), obesity, and dyslipidemia.^1^ Among patients with MetS, cardiovascular disease remains the leading cause of death in those with NAFLD.^1^ Coexisting T2DM and NAFLD significantly raise cardiovascular risk, largely due to atherogenic dyslipidemia marked by high triglycerides, low HDL cholesterol, and small, dense LDL particles.^12^ NAFLD severity—including advanced fibrosis, cirrhosis, and HCC—strongly correlates with diabetes, even when liver enzymes remain normal.^13,14^

 T2DM and NAFLD share a bidirectional, mutually reinforcing relationship: individuals with T2DM have a higher risk of NAFLD, and vice versa. Globally, the incidence of T2DM in individuals with NAFLD reaches 24 cases per 1,000 person-years.^15^ Overweight and obese individuals face nearly triple the risk of NAFLD compared to those with normal weight.^16^ Common pathophysiological mechanisms, such as obesity, dyslipidemia, and insulin resistance (IR), link these conditions.^12^

 Given this close association, clinicians must adopt integrated treatment strategies to manage T2DM and NAFLD together. This review explores the complex interplay between these two metabolic disorders and proposes strategies for their joint prevention and management.

## Global prevalence and incidence

 According to the National Diabetes Statistics Report released by the Centers for Disease Control and Prevention in 2021, approximately 11.6% of Americans have T2DM, while 38% are affected by prediabetes. The burden of both conditions increases substantially with age: among individuals aged 65 and older, the prevalence of diabetes and prediabetes is reported at 29.2% and 48.8%, respectively. Stratified by ethnicity, the highest prevalence of diagnosed diabetes was observed in American Indian and Alaska Native adults (13.6%), followed by non-Hispanic Black (12.1%), Hispanic (11.7%), non-Hispanic Asian (9.1%), and non-Hispanic White adults (6.9%) (CDC, 2021).^17^

 Globally, the prevalence of NAFLD continues to rise, particularly among individuals who are overweight or obese. Current estimates indicate that NAFLD affects nearly 38% of the world’s population. A recent meta-analysis reported a global pooled prevalence of 30.05%, with East Asia exhibiting the highest regional prevalence at 32.31%. Notably, NAFLD prevalence has increased by more than 50%, rising from 25.26% during 1990–2006 to 38.20% between 2016–2019. The more advanced and progressive form of the disease, NASH, has a global prevalence ranging from 5% to 7%. However, this figure increases substantially in individuals with T2DM, in whom the prevalence of NASH is estimated at 37%, with approximately 17% affected by advanced liver fibrosis. Additionally, the pooled incidence of NAFLD is estimated at 48.89 cases per 1,000 persons per year, reflecting a 58% increase compared to earlier estimates from 1994–2006. Males tend to exhibit a higher median prevalence of NAFLD than females (40% vs. 26%; *P* < 0.0001).^18^

## Association between NAFLD and T2Dm

###  Cardiovascular risks

 Individuals with T2DM who also have NAFLD are at significantly elevated risk for CV complications. Studies indicate that the presence of NAFLD increases the risk of developing CV disease by approximately twofold in individuals with T2DM compared to those without NAFLD.^19^ This increased risk is supported by evidence of greater carotid intima-media thickness in patients with NAFLD and T2DM, a surrogate marker of atherosclerosis and predictor of CV events.^20^ Moreover, these individuals often exhibit elevated coronary artery calcium (CAC) scores, which are indicative of subclinical coronary artery disease and correlate with higher risk of adverse cardiac outcomes.^21^ Additionally, patients often present with early left ventricular diastolic dysfunction, reduced myocardial perfusion, and impaired oxygen delivery to cardiac tissue, all of which contribute to ischemic heart disease.^22^ These patients also show diminished myocardial high-energy phosphate metabolism, compromising cardiac contractility and overall function.^23^

###  Microvascular complications

 Beyond macrovascular disease, NAFLD exacerbates diabetic microvascular complications, such as diabetic retinopathy and chronic kidney disease.^24^ These pathological changes are driven by a constellation of harmful mediators—pro-inflammatory, procoagulant, and prooxidant molecules—which contribute to systemic IR, atherogenic dyslipidemia, and hepatic secretion of key proteins including retinol-binding protein-4 (RBP-4), fibroblast growth factor-21 (FGF-21), and fetuin-A.^25^ Notably, fetuin-A impairs insulin signaling by inhibiting insulin receptor tyrosine kinase activity in the liver and skeletal muscle, thereby worsening IR. This contributes to the accelerated onset of microvascular complications in diabetic patients.^25^

###  Shared mechanism

 Advanced stages of NAFLD—including NASH, liver fibrosis, cirrhosis, and HCC—occur more frequently in T2DM populations.^26^ Disease progression from simple steatosis to these advanced forms is driven by worsening metabolic dysfunction.^27^ A predictive model by Bazick et al for diagnosing NASH and advanced fibrosis in NAFLD patients with T2DM achieved 90% specificity and 56.8% sensitivity by integrating BMI, waist circumference, HbA1c, insulin resistance, ferritin, albumin, ALT, and AST.^28^ Elevated inflammatory markers and hyperinsulinemia further link T2DM to increased HCC risk.^29^ These associations highlight the importance of reciprocal screening for NAFLD and T2DM. Non-invasive tools like elastography are increasingly valuable for detecting hepatic fibrosis in these patients.^30^ Although strong associations exist between NAFLD and CV disease, chronic kidney disease, and liver outcomes in T2DM, further research is essential to confirm causality and refine integrated screening and management strategies.

## Pathogenesis

**Figure 1:**
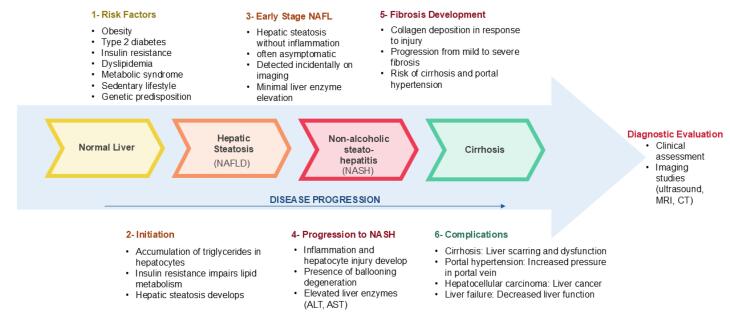
Key risk factors for NAFLD development and progression. This figure highlights the primary risk factors linked to the onset and advancement of NAFLD. Abbreviations: NAFLD, Non-alcoholic fatty liver disease; NASH, Non-alcoholic steatohepatitis; ALT, alanine aminotransferase; AST, aspartate aminotransferase; MRI, Magnetic resonance imaging; CT, Computed Tomography

 NAFLD develops through multiple interacting mechanisms. Excess hepatic lipid accumulates when fatty acid influx from diet, adipose lipolysis, and de novo lipogenesis surpasses disposal via VLDL export and β-oxidation.^31^ Although these pathways initially upregulate to compensate, they eventually fail, leading to hepatocyte lipid retention. Hepatic IR, partly driven by diacylglycerol (DAG)-mediated activation of PKCε, disrupts insulin signaling and aggravates systemic IR in skeletal muscle and adipose tissue.^32^ Inefficient mitochondrial fat oxidation and toxic lipid intermediates further induce hepatocyte injury, oxidative stress, and cytokine-driven necroinflammation—creating a “lipotoxic” environment that drives progression from simple steatosis to NASH and fibrosis.^33^ Figure 1 describes the key risk factors for NAFLD development and progression.

 Recent findings expand this mechanistic view to include broader metabolic and cardiometabolic implications. For example, a paediatric meta-analysis reported that structured lifestyle interventions and targeted supplementation reduce ALT, AST, BMI, and insulin resistance in children with NAFLD. However, the review also revealed major evidence gaps and heterogeneity in trial design, highlighting the need for more robust studies.^34^

## Clinical presentation and diagnostic approaches

 Most patients with NAFLD remain asymptomatic in early stages and report no liver-related symptoms. Hepatomegaly is the most common clinical finding, detected by abdominal palpation. As the disease progresses to NASH or cirrhosis, complications such as portal hypertension become more evident.^34^ Clinicians should suspect NAFLD in patients with MetS components—obesity, hypertension, hyperglycemia, or dyslipidemia—and hepatic steatosis on imaging. Diagnosis relies on exclusion of other causes like alcohol use or hepatotoxic drugs.^28,34^

 After identifying NAFLD, clinicians must determine progression to NASH or fibrosis because these increase complication risk and worsen outcomes. Liver biopsy remains the gold standard for differentiating NAFL from NASH, staging fibrosis, and excluding other chronic liver diseases.^35^ However, its invasiveness, cost, and associated risks—pain, bleeding, and rare complications—limit its routine use.

 Non-invasive tools, such as elastography for liver stiffness assessment, now play a key role in fibrosis evaluation.^36,37^ Clinicians also use scoring systems like NAFLD Fibrosis Score, FIB-4, and APRI, based on clinical and biochemical data.^38^ Table 1 summarizes these serum-based markers.

**Table 1:** Non-invasive Serum biomarkers methods for NAFLD screening ^44^

**Diagnostic panel**	**Description**	**Accuracy **			**Dual cut offs**	**Detection capacity **
		**AUROC score**	**Sensitivity**	**Specificity**		
**Blood based tests **						
HSI	Parameters involved BMI, sex, AST: ALT ratio and T2D presence	0.81 in detecting NAFLD	93.1% 46%	92.4% 92%	HSI < 30- rule out FL HSI > 36- rule in FL	Detect Steatosis but inadequate distinction of severity
FLI	Parameters involved BMI, WC, serum GGT, Triglycerides	0.84 in detecting FL	87% 61%	64% 86%	FLI < 30 rule out FL, FLI ≥ 60 rule in FL	Detect Steatosis but cannot distinguish between grades
Steato Test	Rare biomarkers not done routinely like GGT, total bilirubin, α2m, ApoA-1, haptoglobin, ALT, BMI, total cholesterol, Triglycerides, and glucose adjusted for age, gender, weight and height	0.80 for steatosis > 5%	90% 46%	54% 88%	Steato Test < 0.3 can exclude grade 2–4 steatosis; Steato Test ≥ 0.7 is suggestive of grade 2–4 steatosis	High cost and α2m, ApoA1, haptoglobin are not available in routine examination
K-NAFLD score	Parameters involve sex, WC, SBP and Triglycerides	0.93 in detecting NAFLD	PPV 99% and NPV 72.3%	K-NAFLD < −3.285 rule out NAFLD, K-NAFLD > 0.884 rule in NAFLD	For reference standard NAFLD liver fat score is used instead of biopsy
NAFL screening score	Parameters involved AST: ALT, BMI, fasting plasma glucose, age, uric acid, triglycerides	0.825 for males	79.9%	66.3%	Cut-off of NAFL screening score Male: 33 Female: 29	For reference standard NAFLD liver fat score is used instead of biopsy
		0.861 for females	89.4%	69%		
NFL Score	AST: ALT ratio and T2D presence, metabolic syndrome,	0.86	84% 95%	69% 56%	NFL ≤ 0.640 and ≥ 1.413	Limited availability as insulin level is needed
NAFL risk score	BMI, triglycerides multiply by GGT, AST: ALT ratio, LDL-C, HDL-C, uric acid	0.743 for males	-	-	-	No validation study, Suboptimal gold standard based on ultrasonography
		0.820 for females	-	-		
LAP score	Parameters involve sex, WC and TG	0.79 0.68	93% 86%	34% 50%	LAP score > 30 LAP score > 40	No validation study, Suboptimal gold standard based on ultrasonography
ION	Male: waist-to-hip ratio, triglycerides, ALT and HOMA Female: triglycerides, ALT and HOMA	0.77	81% 60%	56% 82%	ION < 11 ION ≥ 22	No validation study, Suboptimal gold standard based on ultrasonography

α2M, α2-macroglobulin; ALT, alanine aminotransferase; ApoA-1, Apolipoprotein AI; AST, aspartate aminotransferase; AUROC: Area under the receiver-operating characteristics curve; BMI, body mass index; FL, Fatty liver; FLI, Fatty liver index; FSI, Fasting insulin serum; GGT, gamma-glutamyl transferase, HDL-C, high density lipoprotein cholesterol; HOMA, homeostatic model assessment for insulin resistance; HSI, Hepatic steatosis Index; ION, index of nonalcoholic steatohepatitis; LAP, Lipid accumulation product; LDL-C, low-density lipoprotein cholesterol; NFL, NAFLD Liver fat; NAFLD, non-alcoholic fatty liver disease; NPV: Negative predictive value; PPV: Positive predictive value; SBP: Systolic blood pressure; T2D, Type 2 Diabetes mellitus; WC, waist circumference.

 Despite progress, these methods lack the accuracy of biopsy in some cases.^39^ Current guidelines recommend biopsy for patients with high suspicion of NASH or advanced fibrosis, while non-invasive tests guide evaluation and monitoring in low-risk cases.^40-42^ Research is advancing novel tools—biomarkers, metabolomics, and genetic testing—to improve diagnostic precision and enable early risk stratification.^43^

## Current guidance on screening for NAFLD in High-risk patients

 There is increasing recognition of the need for early identification of NAFLD, particularly within primary care settings, given its high prevalence and severity in individuals with MetS and T2DM.^45^ Due to the often-silent nature of the disease, a significant proportion of individuals with undiagnosed NAFLD and advanced fibrosis remain in the community. Detecting these cases early is critical, as no pharmacological treatments are currently approved for NAFLD or NASH.^46^ While routine population-based screening remains controversial—primarily due to concerns about cost-effectiveness, test reliability, biopsy-related risks, and the absence of disease-specific therapies—there is growing consensus that targeted screening of high-risk groups (such as individuals over 50 years of age, and those with T2DM or MetS) is important for prognostic assessment, particularly when advanced fibrosis is suspected.^47,48^

**Figure 2:**
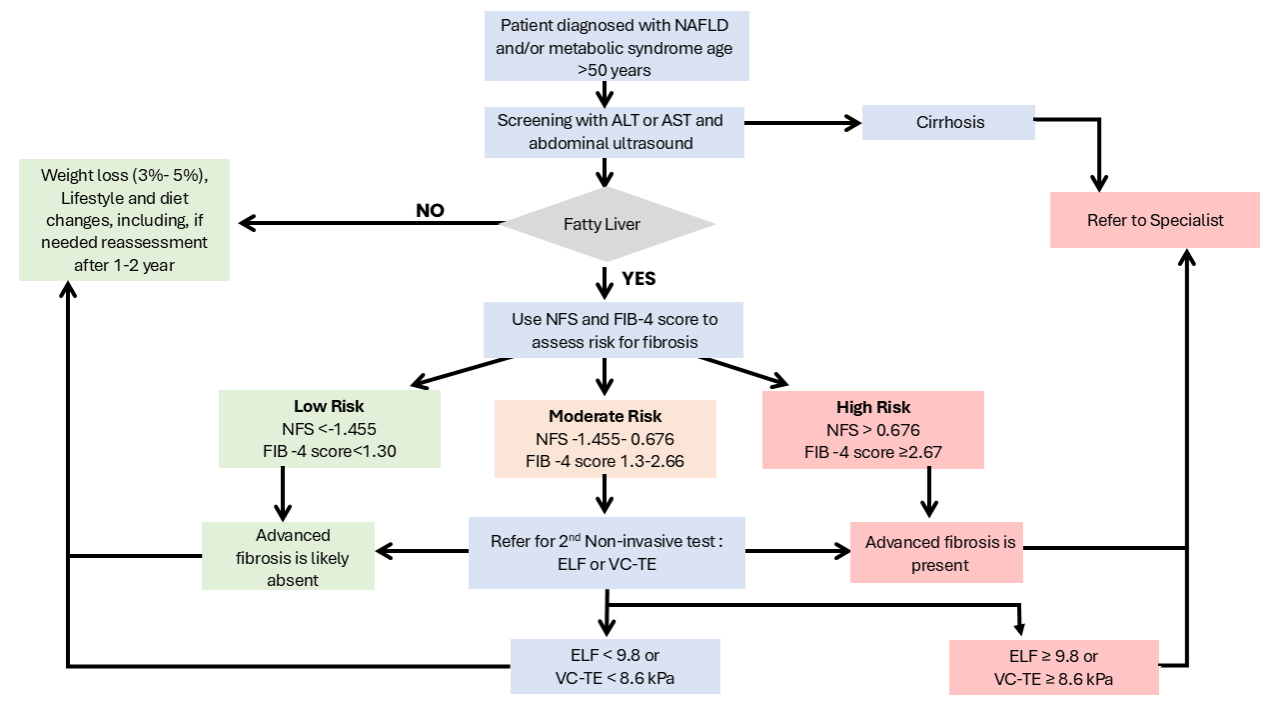
Proposed protocol for NAFLD screening and management. A stepwise approach for the screening and management of non-alcoholic fatty liver disease (NAFLD) is illustrated. The protocol incorporates initial risk stratification followed by non-invasive assessment using either the Enhanced Liver Fibrosis (ELF) test or vibration-controlled transient elastography (VC-TE). Patients are guided through diagnostic and management pathways based on fibrosis risk, aiming for early identification of advanced liver disease and timely intervention

 However, existing clinical guidelines offer divergent recommendations regarding NAFLD screening. The American Association for the Study of Liver Diseases (AASLD) currently discourages routine screening in the general population, irrespective of body mass index (BMI), though it emphasizes the importance of increased awareness among individuals with T2DM.^48^ Similarly, the National Institute for Health and Care Excellence (NICE) in the United Kingdom has not recommended routine screening in its clinical guidelines.^34^ In contrast, the European Association for the Study of the Liver (EASL), along with the European Association for the Study of Diabetes (EASD) and the European Association for the Study of Obesity (EASO), advocates for targeted screening in individuals with MetS or obesity, particularly those meeting specific risk criteria.^49^ Likewise, regional organizations such as the Asian Pacific Association for the Study of the Liver (APASL) and the Korean Association for the Study of the Liver (KASL) also support risk-based screening strategies.^50,51^ Furthermore, screening recommendations vary across professional societies, including those specializing in diabetes, paediatrics, and endocrinology, reflecting ongoing debate and evolving evidence in these subpopulations.^52-54^ Figure 2 outlines a proposed protocol for screening and managing NAFLD, incorporating current international recommendations and risk stratification strategies.

## Lifestyle and pharmacological approaches in NAFLD management

 No anti-diabetic drug currently holds approval for NAFLD treatment. Weight loss and physical activity remain the only consistently effective interventions for improving biochemical markers and histological features—steatosis, inflammation, and fibrosis. Evidence shows physical activity benefits liver health even without significant weight loss,^55,56^ but sustained weight reduction offers the greatest improvements.^57,58^

 RCTs and observational studies confirm that weight loss correlates with better liver biomarkers in NAFLD and NASH.^59^ A ≥ 5% weight loss improves steatosis, while 7–9% reduces inflammation and ballooning. Meaningful fibrosis regression usually requires > 10% reduction.^2,60^ Despite proven benefits, maintaining such weight loss remains challenging, especially in obese and insulin-resistant patients.

 Because NAFLD and T2DM share epidemiological and metabolic pathways, researchers have explored antidiabetic agents—such as insulin sensitizers, GLP-1 receptor agonists, and SGLT2 inhibitors—for NAFLD treatment. Many show promise in reducing steatosis and inflammation, but large-scale, long-term trials are needed before recommending them clinically.^61-63^

###  Metformin

 Metformin, a widely prescribed biguanide for T2DM, reduces hepatic glucose production and improves insulin sensitivity, thereby lowering blood sugar levels without causing weight gain.^64,65^ It primarily activates AMP-activated protein kinase (AMPK) in hepatocytes, which suppresses gluconeogenesis and decreases hepatic lipid accumulation.^66^ Additionally, metformin inhibits de novo lipogenesis and promotes fatty acid oxidation, reducing liver fat content.^67^ These actions make metformin effective for glycemic control in T2DM and for addressing metabolic dysfunctions linked to NAFLD and MetS.^68,69^

 Metformin also modulates gut microbiota by promoting beneficial bacteria and reducing harmful microbes, potentially enhancing gut barrier function and preventing harmful substances from entering the bloodstream—key factors in NAFLD pathogenesis.^68,69^ Studies, including those by Mazza et al, indicate that metformin may benefit NAFLD management even in non-diabetic patients, especially when combined with a hypocaloric diet and weight control.^70^

###  Thiazolidinediones (TZDs)

 TZDs primarily manage T2DM by activating peroxisome proliferator-activated receptor-gamma (PPAR-γ) receptors. These nuclear receptors, present in adipose tissue, liver, skeletal muscle, and the gastrointestinal tract, regulate energy balance, lipid storage, and glucose metabolism. By activating PPAR-γ, TZDs promote adipogenesis to lower free fatty acids, improve insulin sensitivity in the liver, adipose tissue, and muscles, enhance glucose uptake in peripheral and splanchnic regions, and reduce hepatic glucose production.^71,72^

 Troglitazone, the first TZD, improved liver histology in NASH patients but was later withdrawn due to liver toxicity.^73^ Newer TZDs—pioglitazone and rosiglitazone—remain approved and have not shown a similar risk of hepatotoxicity, even with extensive clinical and post-marketing exposure.^74^ Both agents improve histological and biochemical features of NASH, reinforcing the role of insulin resistance in its pathogenesis.^75^

###  Pioglitazone

 Clinicians recommend pioglitazone, a TZD derivative and PPAR-γ agonist, for T2DM.^76^ At low doses, it reduces inflammation and fibrosis in NAFLD, increases adiponectin, and enhances insulin sensitivity. Pioglitazone improves glucose and lipid metabolism, lowers triglycerides and small, dense LDL, raises HDL, and normalizes free fatty acids, reversing atherogenic dyslipidemia.^74^ Studies show pioglitazone improves liver histology and biochemical markers, while discontinuation worsens NASH.^77^ Mahady et al reported improvements in steatosis, inflammation, ballooning, and fibrosis.^78^

 Pioglitazone promotes adipogenesis and can cause weight gain, especially with long-term use.^79^ Meta-analyses confirm higher weight and BMI in non-diabetic patients vs. placebo, consistent with earlier studies.^78-80^ Weight gain in diabetic NAFLD patients was not statistically significant and may result from increased fat and fluid retention.^81^

 Pioglitazone slows progression from prediabetes to T2DM^82,83^ and reduces CVD risk in both diabetic and non-diabetic individuals.^83,84^ It also slows atherosclerosis, improves diastolic function, and reduces epicardial fat.^85,86^ In Sanyal’s trial of 247 non-diabetic NASH patients, vitamin E—but not pioglitazone—showed significant NASH improvement, although both improved LFTs.^87^ Cusi et al observed NASH resolution in 51% of pioglitazone-treated patients vs. 19% placebo (*P* < 0.001).^88^

 Use of pioglitazone in non-diabetic patients is debated. Tang and Ferwana linked long-term use to bladder cancer,^89,90^ though Korhonen disputed this claim.^91^ Meta-analyses indicate risks of heart failure^92^ and fractures,^93^ while edema is more common when combined with insulin.

 European guidelines (EASL, EASD, EASO) recommend pioglitazone for treating NAFLD in patients with T2DM and occasionally off-label for non-diabetics. In contrast, American (AASLD) and UK (NICE) guidelines restrict its use to diabetic patients with NAFLD.^94^ Recently, the ADA included pioglitazone in its recommendations for drug classes that improve NASH in diabetic patients.^95^

####  Rosiglitazone

 The FDA approved rosiglitazone for T2DM, but Europe withdrew it in 2010 and the U.S. restricted its use in 2011 due to CV safety concerns, despite favourable NAFLD outcomes.^96^ In a single-arm study of 30 NASH patients with impaired glucose tolerance or T2DM, 8 mg/day for 48 weeks resolved NASH in 45% and significantly reduced ALT.^97^ A larger trial of 68 patients showed improved glycemic control and liver enzymes after 24 weeks, with a mean weight gain of 2.6 ± 2.4 kg.^98^

 The FLIRT trial reported that rosiglitazone improved steatosis in 47% vs. 16% with placebo and normalized transaminases, but prolonged use did not improve fibrosis and caused weight gain.^99,100^ A subsequent study of 135 NASH patients found no significant histological benefit from adding metformin or losartan; metformin slightly reduced weight gain, but not significantly.^101^ Further FLIRT analysis showed increased hepatic PPARγ and pro-inflammatory gene expression, raising safety concerns.^102^ These findings, combined with CV risk and weight gain, limit rosiglitazone’s role in NAFLD management.

####  Lobeglitazone

 Lobeglitazone, a newer TZD and dual PPARγ/partial PPARα agonist, is marketed in Korea as Duvie® for diabetes.^103^ In a mouse model of diet-induced NAFLD, 4 weeks of lobeglitazone improved glucose regulation, steatosis, and lipid levels by upregulating fatty acid β-oxidation genes and downregulating lipogenesis and gluconeogenesis genes.^104^ Human data are limited. In a single-arm trial of 50 T2DM patients with NAFLD (CAP > 250 dB/m), 0.5 mg lobeglitazone for 24 weeks modestly but significantly reduced steatosis, improved glycemic control, and decreased atherogenic dyslipidemia.^105^

###  Sodium–glucose linked transporter-2 (SGLT2) inhibitors

 SGLT2 inhibitors improve NAFLD via multiple mechanisms: better glycemic control, reduced visceral fat, increased adiponectin, lower uric acid, reduced oxidative stress and inflammation, and weight loss. They also provide cardiorenal protection.^106^ These agents block renal glucose reabsorption, causing glycosuria, and are effective in heart failure and CKD, even without diabetes.^107^ They improve mitochondrial function and increase β-hydroxybutyrate levels but carry risks of genitourinary infections and rare ketoacidosis.^106^ Studies show SGLT2 inhibitors lower AST more effectively than pioglitazone, though their advantage over GLP-1 RAs is not significant.^108^ GLP-1 RAs remain effective but limited by GI side effects and injection requirements.

###  Glucagon-like peptide-1 (GLP-1) RAs

 GLP-1 RAs reduce major adverse cardiovascular events (MACEs) and slow kidney disease progression in T2DM.^109^ These agents improve insulin sensitivity, suppress hepatic glucose production, and reduce hepatic fat via decreased lipogenesis and increased fatty acid oxidation.^110,111^ They also promote weight loss and are approved for obesity treatment.^112^ FDA-approved agents include exenatide, lixisenatide, liraglutide, semaglutide, dulaglutide, and albiglutide, administered daily or weekly.^113^ Long-term use lowers HbA1c by 0.8–1.9%, with long-acting forms offering superior glycemic control.^114,115^

 Liraglutide is the most studied GLP-1 RA for NAFLD. It improves insulin sensitivity, reduces liver fat, and lowers fibrosis risk (3.1% vs. 6.1%) in T2DM patients.^116^ A Phase II trial showed daily 1.8 mg liraglutide for 48 weeks improved histology and resolved NASH in 39% vs. 9% with placebo (*P*= 0.019).^117^

 Semaglutide demonstrated safety and improved glycemic control in compensated NASH cirrhosis but did not significantly improve fibrosis after 48 weeks. Non-invasive measures and MRI-PDFF showed fat reduction.^118^

 Tirzepatide, a dual GIP/GLP-1 agonist, reduced liver fat in the SURPASS-3 substudy across all doses.^119^

###  α-Glucosidase inhibitors

 Agents like acarbose, miglitol, and voglibose slow carbohydrate digestion and modestly lower glucose but have limited NAFLD data.^120^ A 24-week mouse study combining ezetimibe and acarbose improved histology.^121^ In a human trial of 17 T2DM patients with NASH, miglitol (150 mg/day, 12 months) reduced BMI, ALT, and improved steatosis and inflammation, but not fibrosis or ballooning.^122^ Acarbose appears safe in cirrhosis^123,124^ and reduces CV events and hypertension in prediabetes,^125^ though it can mildly raise ALT and, rarely, cause hepatotoxicity.^126^

## Recent evidences and pipeline studies

 Recent evidence strengthens the role of IR and metabolic dysfunction in NAFLD and highlights its bidirectional relationship with T2DM. Oh et al reported that NAFLD independently increases the risk of heart failure and CV mortality in T2DM patients.^127^ Zhang et al identified triglyceride-glucose (TyG) indices—especially TyG-WWI—as strong predictors of all-cause and CV mortality in MASLD with diabetes or prediabetes.^128^ Novel biomarkers, including the TG/APOA1 ratio^129^ and TyG-BMI index,^130^ show superior diagnostic value for detecting NAFLD in T2DM patients. Nontraditional lipid markers, such as the atherogenic index of plasma (AIP) and residual cholesterol (RC), outperform traditional lipid panels in predicting abnormal glucose metabolism.^131^ Wu et al also linked elevated thyroid autoantibodies to a higher risk of NAFLD in T2DM.^132^ In paediatric NAFLD, lifestyle changes and nutritional supplements significantly improved liver enzymes and insulin resistance, emphasizing early intervention benefits.^133^ Collectively, these findings underscore the metabolic and cardiovascular impact of NAFLD and the need for early detection and integrated care.

 In March 2024, the US FDA approved resmetirom as the first drug specifically for NAFLD treatment.^134^ Previously, no FDA-approved therapy existed for NAFLD, including antidiabetic agents. Although clinicians frequently used drugs like metformin, thiazolidinediones, SGLT2 inhibitors, and GLP-1 RAs off-label to manage metabolic dysfunction in NAFLD, none held official approval for this indication. Numerous studies remain ongoing, and over 10 active trials are investigating antidiabetic drugs for NAFLD treatment (Supplementary file 1, Table S1).^135^

 Several clinical trials aim to identify effective treatments for NAFLD and NASH. A trial measuring hepatic mitochondrial fluxes will compare pioglitazone to placebo, with results expected by March 2027.^136^ AIM 2 is testing low-dose pioglitazone in NASH, with completion anticipated in August 2027.^137^ Another study is evaluating efinopegdutide versus Semaglutide and placebo in precirrhotic NASH, with results expected in February 2026.^138^ The WAYFIND trial is assessing semaglutide combined with cilofexor and firsocostat in cirrhotic NASH patients, reporting in December 2024.^139^ Additional trials with dapagliflozin, bempedoic acid, and henagliflozin are underway, with completion dates between December 2024 and November 2026.^140-142^ Upcoming trials will also compare empagliflozin with pioglitazone in non-diabetic NASH and examine SGLT2 inhibitors in MASLD, both launching later in 2024.^143^ These studies reflect a comprehensive effort to develop targeted therapies for NAFLD and NASH across different disease stages.

Table S2 summarizes key studies on pioglitazone in NAFLD.

## Challenges and future directions

 Managing NAFLD and T2DM remains challenging despite increasing recognition of their interconnection. Clinicians often miss NAFLD in T2DM patients because it typically remains asymptomatic until advanced stages. The lack of standardized screening protocols further complicates detection and management. Current pharmacological options—metformin, thiazolidinediones, SGLT2 inhibitors, and GLP-1 RAs—show promise but do not fully address NAFLD pathogenesis and exhibit inconsistent efficacy. Researchers must also clarify the long-term safety and potential adverse effects of these drugs across diverse populations.

 This review has limitations. Included studies vary widely in design, sample size, and diagnostic criteria, limiting comparability and generalizability. Long-term outcomes, such as fibrosis regression and cardiovascular risk reduction, remain underexplored due to short follow-up durations. Most pharmacological evidence relies on off-label use and preliminary findings rather than robust, large-scale randomized controlled trials. Additionally, publication bias and the exclusion of unpublished or ongoing trial data may have influenced the completeness of this review.

 Future research should focus on developing precise diagnostic tools and early-stage biomarkers, especially for T2DM patients. Personalized medicine approaches using genetic, epigenetic, and metabolic profiling could enable tailored therapies. Large-scale, long-term clinical trials are essential to confirm the safety and efficacy of both existing and emerging treatments. Integrating lifestyle interventions such as dietary modifications and physical activity with pharmacotherapy offers additional benefits. Progress will require close collaboration among endocrinologists, hepatologists, and primary care providers. Ultimately, a multidisciplinary approach and sustained research are critical to reducing the global burden of NAFLD and T2DM.

## Conclusion

 NAFLD and T2DM are closely linked metabolic disorders that demand a unified, comprehensive management approach. There is an urgent need to raise awareness, promote early detection, and implement evidence-based strategies to alleviate the burden of these comorbidities. A holistic approach that considers the complex relationship between NAFLD and T2DM can help healthcare providers improve patient outcomes and reduce the societal impact of these growing public health challenges. Due to its asymptomatic nature, NAFLD often remains undiagnosed. Early screening and risk stratification are essential to prevent progression to advanced liver disease.

## Competing Interests

 The authors declare that they have no competing interests.

## Ethical Approval

 Not applicable.

## Supplementary Files


Supplementary file 1 contains Table S1 and Table S2.
