# Supplementary material for: Diabetes and NAFLD: A Synergistic Threat to Metabolic Health
Source: Adv Pharm Bull. 2025 Oct 18;15(4):766–78. doi: 10.34172/apb.025.44094 (PMC12980267; doi:10.34172/apb.025.44094)
Supplement: Supplementary file 1 — contains Table S1 and Table S2. [file apb-15-766-s001.pdf]

Adv Pharm Bull. 2025;15(4):766-S1
doi: 10.34172/apb.025.44094
https://apb.tbzmed.ac.ir

Supplementary Information

Article title: Diabetes and NAFLD: A Synergistic Threat to Metabolic Health

Table S1: Pipeline studies for antidiabetic drugs in treatment of NAFLD (retrieved from (Clinical trial, 2024[135])

Study Title	Status	Sponsor	Phase	Study Type	Estimated Completion Date
Quantifying Hepatic Mitochondrial Fluxes in Humans	Recruiting	Pioglitazone vs. Placebo	4	Interventional	March 2027
Low-Dose Pioglitazone in Patients with NASH (AIM 2)	Recruiting	Pioglitazone vs. Placebo	2	Interventional	August 2027
A Clinical Study of Efinopegdutide in Participants with Precirrhotic Nonalcoholic Steatohepatitis (NASH) (MK-6024-013)	Recruiting	Efinopegdutide vs. Semaglutide vs. Placebo	2	Interventional	February 2026
Study of Semaglutide, and Cilofexor/Firsocostat, Alone and in Combination, in Adults with Cirrhosis Due to Nonalcoholic Steatohepatitis (NASH) (WAYFIND)	Active, not recruiting	Semaglutide vs. FDC of Cilofexor & Firsocostat vs. Placebo	2	Interventional	December 2024
Dapagliflozin in Type 2 Diabetes Mellitus Patients (T2DM) with Nonalcoholic Fatty Liver Disease (NAFLD)	Recruiting	Dapagliflozin vs. Placebo	4	Interventional	December 2024
Effect of Bempedoic Acid on Liver Fat in Individuals with Nonalcoholic Fatty Liver Disease and Type 2 Diabetes (B-LIFT)	Recruiting	Bempedoic acid	NA	Interventional	December 2024
Effect of Henagliflozein on Hepatic Fat Content in Patients with T2DM and NAFLD (HHTN)	Recruiting	Bempedoic acid	4	Interventional	November 2026
Comparative Clinical Study Between Empagliflozin Versus Pioglitazone in Non-diabetic Patients with Non-alcoholic Steatohepatitis	Not yet recruiting	Pioglitazone vs. Empagliflozin	3	Interventional	November 2024
The Impact of SGLT2 -I on Metabolic Dysfunction -Associated Steatotic Liver Disease In Patients With Type 2 Diabetes Mellitus	Not yet recruiting	SGLT-2 inhibitor	3	Interventional	October 2024

Table S2: Important studies of Pioglitazone in NAFLD patients

Study	Intervention & comparator	No. of patients	Dose	Treatment duration	Patient with NASH resolution (%)	People With Fibrosis Improvement, %*
Yoneda et. al. 2022 [144]	- Pioglitazone<br>- Pioglitazone + Tofogliflozin	32	15-30 mg/day	24 weeks	51%	Not reported
Cusi et. al. 2016 [88]	- Pioglitazone<br>- Placebo	101	45 mg/day	72 weeks	32%	41%
Huang et. al.; 2021[145]	- Pioglitazone<br>- Placebo	90	30 mg/day	24 weeks	16%	1%
Bril et. al.; 2019 [146]	- Pioglitazone+ Vitamin E<br>- Vitamin E	105	45 mg/day	72 weeks	31%	22%
Sanyal et. al. 2010 [87]	- Pioglitazone<br>- Vitamin E	247	30 mg/day	96 weeks	26%*	13%
Aithal et. al.; 2008 [147]	- Pioglitazone<br>- Placebo	74	30 mg/day	50 weeks	Not reported	9%
Belfort et. al.; 2006[148]	- Pioglitazone<br>- Placebo	55	45 mg	24 weeks	Not reported	13%

* Not significant
